# Physical and Mental Health as Biopsychosocial Correlates and Pathways Associated With Symptom Severity in Irritable Bowel Syndrome: A Cross‐Sectional Structural Equation Modeling Study

**DOI:** 10.1111/nmo.70300

**Published:** 2026-04-02

**Authors:** Hannah B. Lindsell, Maura Corsetti, A. M. Darie, Daniele Magistro, A. Mohanan, Gemma E. Walton, Neil C. Williams

**Affiliations:** ^1^ Department of Sport, Health and Performance Enhancement (SHAPE) Research Centre, Department of Sport Science Nottingham Trent University Nottingham UK; ^2^ NIHR Nottingham Biomedical Research Centre Nottingham University Hospitals NHS Trust UK, School of Medicine Nottingham UK; ^3^ Department of Health Sciences, Faculty of Environmental and Life Sciences University of Southampton Southampton UK; ^4^ Department of Food and Nutritional Sciences The University of Reading Reading UK

**Keywords:** anxiety, biopsychosocial, depression, disorder of gut‐brain interaction, irritable bowel syndrome, physical health, structural equation modeling

## Abstract

**Introduction:**

Irritable bowel syndrome (IBS) is a heterogenous disorder of gut‐brain interaction, characterized by complex interaction between gastrointestinal symptoms, psychological distress and physical functioning. While physical activity is increasingly recommended as part of IBS management, the pathways through which physical and mental health relate to symptom severity remain incompletely understood.

**Objective:**

To explore the interrelationships between physical activity, physical health, mental health and IBS symptom severity using Structural Equation Modeling (SEM) within a biopsychosocial framework, in adults with IBS.

**Methods:**

In this cross‐sectional exploratory study, 106 adults with a clinician‐confirmed diagnosis of IBS (Rome IV criteria) completed validated questionnaires assessing IBS severity (IBS‐SSS), physical activity (GPAQ), anxiety (GAD‐7), depression (PHQ‐8), and health‐related quality of life (SF‐36). Bivariate correlations were examined, followed by SEM to investigate relationships between latent constructs of physical health and mental health on IBS symptom severity.

**Results:**

Participants (mean age: 45 ± 14 years; 82% female) reported high IBS symptom severity (333 ± 89) and wide‐ranging physical activity levels (mean: 3382 ± 4482 MET‐min/week). Bivariate analysis showed significant correlations between IBS symptom severity and anxiety (*r* = 0.235, *p* < 0.05), depression (*r* = 0.240, *p* < 0.05) and multiple physical health domains, including pain (*r* = 0.454, *p* < 0.001). In the SEM physical health demonstrated a large but non‐significant association with IBS symptom severity (*β* = 0.68, *p* = 0.065), while mental health showed no statistically significant direct path (*β* = 0.19, *p* = 0.261). Physical activity was significantly associated with physical health (*β* = 0.22, *p* < 0.001) and physical and mental health latent constructs were strongly correlated (*β* = 0.72).

**Conclusion:**

In this exploratory SEM study physical and mental health were closely interrelated and associated with IBS symptom severity at the correlational level, although direct paths in the SEM did not reach conventional statistical significance. Physical activity was linked to physical health, suggesting a potential indirect pathway influence symptom severity. These findings support a biopsychosocial perspective and highlight the need for integrated, personalized IBS management approaches, while underscoring the important of larger, longitudinal studies to clarify causal pathways.

## Plain Language Summary

This study explored how physical activity, physical health and mental health relate to symptom severity in adults with Irritable Bowel Syndrome (IBS). A total of 106 adults with clinically diagnosed IBS completed questionnaires measuring IBS symptoms, physical activity levels, anxiety, depression and quality of life. The results showed that more severe IBS symptoms were associated with higher anxiety and depression and poorer physical health. Physical activity was linked to better physical health, and physical and mental health were strongly related to each other. Overall, the findings support the idea that IBS symptoms are influenced by both physical and psychological factors, highlighting the importance of a biopsychosocial approach to IBS management.

## Key Points

1. IBS symptom severity is not just about the gut, it is associated with both physical and mental health factors, supporting a biopsychosocial perspective.
2. Staying active was linked to better physical functioning, while poorer physical health was correlated with greater IBS severity.
3. Both physical health and mental health were strongly interrelated and may represent important factors to consider in IBS management.

## Graphical abstract

IBS is not just about the gut, both physical and mental health influence symptoms. Staying active and maintaining good physical wellbeing is linked to milder IBS symptoms. Supporting both physical health and mental wellbeing can ease IBS severity and improve quality of life.

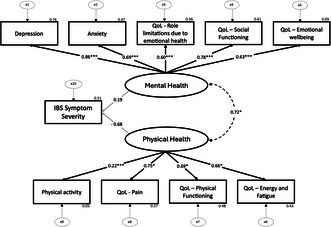

**Abbreviations**
ANOVA: Analysis of Variance
DGBI: Disorder of Gut‐Brain Interaction
GAD‐7: Generalized Anxiety Disorder‐7
GBA: Gut‐brain axis
GPAQ: Global Physical Activity Questionnaire
HRA: Health Research Authority
HRQoL: Health related quality of life
IBS: Irritable Bowel Syndrome
IBS‐SSS: IBS Severity Scoring System
PHQ‐8: Patient Health Questionniare‐8
PPI: Patient and public involvement
QoL: Quality of life
SF‐36: Health related Quality of Life

## 1 Introduction

Irritable Bowel Syndrome (IBS) is a heterogeneous disorder of gut‐brain interaction (DGBI) diagnosed using the Rome IV criteria [1]. It is characterized by abdominal pain or discomfort and altered bowel habits [2] and frequently co‐occurs with psychiatric conditions such as anxiety and depression [3]. Affecting ~3%–5% of the global population [4, 5], IBS represents a complex, multifactorial condition with an etiology that remains poorly understood. This complexity has long supported a biopsychosocial understanding of IBS [6, 7], emphasizing dynamic interactions between physiological processes, psychological functioning and lifestyle, supporting an integrative care approach.

As a chronic condition underpinned by interacting biological, psychological and social factors, IBS care priorities have been long guided towards integrative management models based on this biopsychosocial framework, including interventions targeting lifestyle changes, dietary modification and behavioral factors [8, 9, 10, 11, 12, 13]. Growing interest in the gut microbiome has highlighted potential mechanisms linking lifestyle behaviors, including physical activity to IBS symptom severity, with evidence suggesting physical activity may relieve IBS symptoms via modulation of the gut microbiome in IBS; however, these mechanisms are beyond the primary scope of the present study [14, 15, 16, 17, 18, 19]. Consequently, treatment strategies increasingly combine pharmacological and non‐pharmacological interventions, including dietary modification, psychological therapies, and lifestyle changes [20, 21].

Central to this integrative perspective is the gut‐brain axis (GBA), a bidirectional network involving the enteric nervous system, vagus nerve, and central nervous system, which plays a pivotal role in IBS pathophysiology [22]. Psychological factors (stress, anxiety, and depression) can influence the GBA, potentially exacerbating IBS symptoms through changes in gastrointestinal motility, visceral hypersensitivity, and immune function [23, 24, 25]. Anxiety in particular is highly prevalent in IBS populations and has been shown to independently contribute to symptom severity, often co‐occurring with depressive symptoms [26, 27, 28, 29]. Taken together, these findings within existing literature reinforce the rationale for integrative interventions targeting both physiological and psychological dimensions of the disorder, with lifestyle behaviors such as physical activity serving as interventions that simultaneously address multiple domains of health.

Within this context, exercise has emerged as a valuable adjunct therapy [21]. Physical activity, is defined as any bodily movement produced by the skeletal muscles that results in energy expenditure [30], and as a lifestyle intervention has gained increasing recognition for its capacity to improve both physical and mental health outcomes in IBS patients [12, 21, 31]. However, the type, intensity and tolerability of physical activity may be particularly relevant in IBS populations for whom symptoms and psychological distress can influence engagement in, and response to, exercise.

Despite the known benefits of physical activity [11, 12, 13, 31], many IBS patients remain sedentary, often due to barriers such as fatigue, symptom‐related fear, uncertainty about suitable exercise, and limited awareness of its benefits [32, 33, 34]. Qualitative studies reveal the nuanced and individualized nature of these barriers and motivations. One study found that individuals modify their physical activity in response to symptoms, life situation, and self‐image [34], while Groenendijk et al. [35] highlighted that female IBS patients experienced both benefits (e.g., symptom relief and improved wellbeing) and challenges (e.g., embarrassment, anxiety, and symptom exacerbation) related to physical activity, depending on timing, type, and intensity. Although physical activity is increasingly recognized as a multi‐domain intervention, the mechanisms through which it influences IBS symptom burden remains incompletely understood. Proposed pathways include modulation of the gut microbiome, enhanced psychological wellbeing and health‐related QoL [21] mediated by anxiety, depression and gut specific psychological distress [36].

Together, these studies underscore the need for personalized and flexible physical activity approaches alongside a greater understanding of the multifaceted role that physical activity can play in supporting IBS management. The protective effects of physical activity on mental health are well established [37, 38, 39, 40]. A meta‐analysis of 49 studies in 266,939 participants [41] reported more physically active individuals to have a 17% reduced risk of developing depression, irrespective of age or geographical region. Importantly, the intensity of exercise plays a critical role in mediating its antidepressant effects.

Structural Equation Modeling (SEM) provides a robust, theory‐driven framework to examine these relationships by simultaneously modeling direct and indirect pathways while accounting for bidirectional and latent constructs representing broader physical and mental health domains [36]. SEM research in IBS demonstrates that the impact of IBS symptom severity on quality of life (QoL) is mediated by gastrointestinal (GI)‐specific anxiety, depressive symptoms, and somatic symptom burden [36]. These findings support the rationale for targeting psychological pathways in IBS treatment and position physical activity as a multi‐domain intervention, addressing both physical and mental health outcomes.

Therefore, using SEM, the aim of this model‐based study is to examine the complex interrelationships between physical health, mental health, QoL, and symptom severity in individuals with IBS, within a biopsychosocial framework. Latent constructs of mental health were derived from validated measures of anxiety, depression, emotional wellbeing, social functioning and role limitation due to emotional health, while physical health was derived from physical activity, physical functioning, pain and energy/fatigue. This model aimed to clarify how these physical and mental health domains contribute to variations in IBS symptom severity, with the goal of informing personalized, integrative IBS care.

## 2 Materials and Methods

### 2.1 Study Design and Participants

This cross‐sectional exploratory study investigated relationships between physical activity, IBS symptoms and psychiatric comorbidities in IBS patents. Patients were recruited from a single outpatient clinic of a referral centre in Nottingham, UK (Nottingham University Hospital NHS Trust). 106 adults with a clinical diagnosis of IBS meeting the Rome IV criteria [1] and knowledge of their IBS subtype based on stool consistency: IBS‐C, predominant constipation (≥ 25% hard stools, < 25% loose stools), IBS‐D predominant diarrhea (≥ 25% loose stools, < 25% hard), IBS‐M, for mixed symptoms (≥ 25% of stools are both hard and loose) and IBS‐U for un‐subtyped (insufficient stool consistency to meet aforementioned criteria) volunteered to participate in this study. Participants were excluded if they had other known gastrointestinal conditions, such as inflammatory bowel disease (IBD), coeliac disease, Crohn's disease or colorectal cancer. The sample size was determined pragmatically based on recruitment feasibility within this specialist outpatient clinic, rather than by a priori power calculation, accordingly analyses were conducted with an exploratory, hypothesis‐generating aim [1].

### 2.2 Questionnaires

The IBS Severity Scoring System (IBS‐SSS) was used to evaluate and quantify gastrointestinal symptom severity among IBS patients [42]. This five‐item measure assesses abdominal pain intensity, abdominal pain frequency (calculated as the number of days with pain in the past 10 days multiplied by 10), bloating, dissatisfaction with bowel habits, and the impact of symptoms on daily life. Each item is scored on a scale from 0 to 100, with higher scores indicating more severe symptoms. The total score ranges from 0 to 500, with severity classified as healthy (< 75), mild (75–174), moderate (175–299) and severe (≥ 300).

The habitual level of physical activity for each patient was measured using the validated and standardized self‐report Global Physical Activity Questionnaire (GPAQ) [43]. The GPAQ, developed by the World Health Organization (WHO), assessed physical activity across three domains: work, transport and recreation. GPAQ reports the frequency and duration of moderate‐ and vigorous‐intensity activities, in addition to daily sedentary time. Data was processed using the WHO scoring guideline to calculate total physical activity in MET‐minutes per week.

Generalized Anxiety Disorder (GAD‐7) is a 7‐item validated screening questionnaire to measure clinical signs of generalized anxiety disorder [44] over the previous 2‐weeks. Each item was rated on a four‐point Likert scale (0 = not at all to 3 = nearly every day), with total scores ranging from 0 to 21. Higher scores were indicative of greater anxiety severity, with thresholds of 0–4 for no anxiety, 5–9 for mild anxiety, 10–14 for moderate anxiety, 15–19 for moderately severe anxiety, and ≥ 20 for severe anxiety. A score of ≥ 10 is commonly used to identify clinically significant anxiety. For the Structural Equation Modeling (SEM), scores were inverted so that higher scores indicate lower anxiety, therefore better mental health, consistent with the directionality of the other mental health indicators.

Depressive symptoms were assessed using the validated 8‐item Patient Health Questionnaire (PHQ‐8) [45]. Each item is rated on a four‐point Likert scale (0 = not at all to 3 = nearly every day), with total scores ranging from 0 to 24, with higher score indicating greater severity of depressive symptoms. Depression severity was classified as: None (0–4), Mild [5, 6, 7, 8, 9], Moderate [10, 11, 12, 13, 14], Moderately Severe [15, 16, 17, 18, 19], and Severe [20, 21, 22, 23, 24]. The validated cutoff for clinically significant depression is a PHQ‐8 score ≥ 10. For the SEM, scores were inverted so that higher scores indicate lower depressive symptoms and therefore better mental health.

Health related quality of life (HRQoL) was assessed using the 36‐item Short Form Health Questionnaire (SF‐36) [46], covering nine domains: Physical Functioning (PF), Role Limitations due to Physical Health (RL‐PH), Role Limitations due to Emotional Health (RL‐EH), Energy/Fatigue (E/F), Emotional Well‐Being (EWB), Social Functioning (SF), Pain (P), General Health (GH), and Health Change (HC). Scoring follows two steps: responses are recoded using a predefined key, with scores ranging from 0 to 100, with higher scores indicating better health status. Then, scores within each domain are averaged to calculate the final subscale scores.

### 2.3 Ethical Considerations

The study was approved on 01.03.2024 by the Health Research Authority (HRA), ref. number 24/SC/0045, and adhered to the ethical principles of the Helsinki declaration. Verbal and written informed consent were obtained from all participants before any study procedures commenced. No monetary or other incentives were provided for participation. Participants could withdraw from the study at any time without providing reason and without any repercussions. Confidentiality of participation was maintained, with data access exclusively limited to the research team members. Participants' personal identity was not revealed during data collection, analysis, presentations, and publications.

### 2.4 Statistical Analysis

Data were analyzed using IBM SPSS (version 28) and IBM SPSS AMOS (version 28). A Pearsons bivariate correlation was conducted using IBM SPSS (version 28; IBM Corporation, Somers, NY, USA) to examine the relationships between IBS symptom severity, perceived mental health (anxiety and depression), QoL and physical activity levels. A correlation matrix summarizes the relationship (Table 3).

**FIGURE 1:**
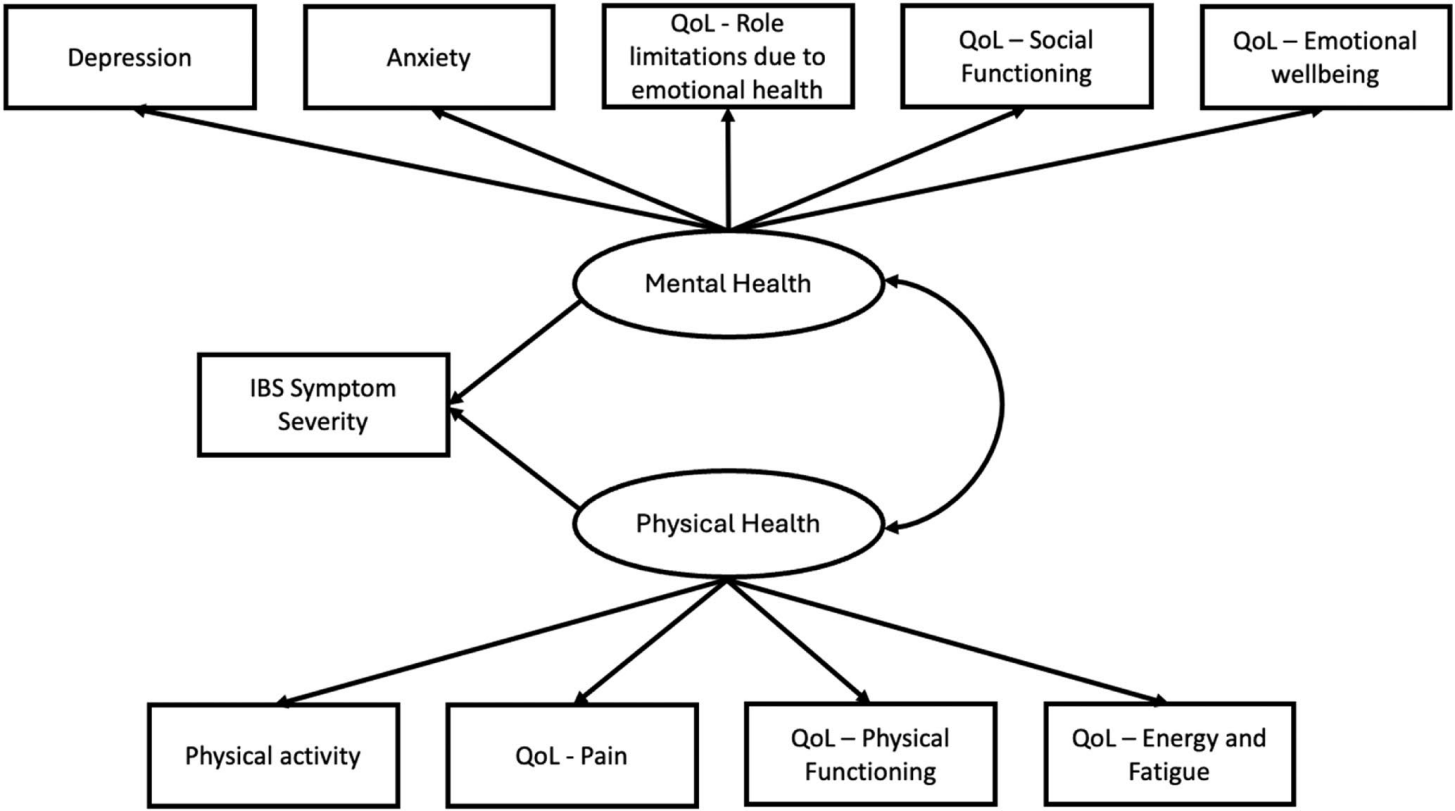
Conceptual and Hypothesized Model (incl. all participants) of the associations between IBS symptom severity, physical health variables and mental health variables.

To further investigate these connections, Structural Equation Modeling (SEM) was conducted using IBM SPSS AMOS (version 28; IBM Corporation, Somers, NY, USA). A hypothesized model was developed (Figure 1) based on established biopsychosocial frameworks, prior SEM studies in IBS populations and evidence linking physical activity, mental health and QoL to IBS [6, 36, 47, 48, 49]. The model development was informed by both the existing literature and clinical observations, iteratively refined to reflect theoretically plausible relationships. The model examined the relationships between physical activity, perceived mental health, physical health, and IBS symptom severity. Two latent variables were defined:

- Mental Health: Indicated by GAD‐7 (anxiety), PHQ‐8 (depression), and relevant SF‐36 domains (role limitations due to emotional health and social functioning)
- Physical Health: Indicated by GPAQ (physical activity) and relevant SF‐36 domains (e.g., pain, physical functioning and energy/fatigue). Physical activity was included as an indicator of physical health to reflect functional capacity and overall physical functioning, rather than behavior alone.

Model fit was evaluated using standard indices: the Tucker‐Lewis Index (TLI), Comparative Fit Index (CFI), and Root Mean Square Error of Approximation (RMSEA).

## 3 Results

### 3.1 Participant Characteristics

This study of included 106 participants (Female: 87, Male: 19) and the distribution of subtypes was IBS‐C (34%), IBS‐D (28.3%), and IBS‐M (37.7%). IBS severity was categorized based on the IBS Severity Scoring System (IBS‐SSS), with patients classified as having mild (7.5%), moderate (28.3%), or severe (64.2%) IBS. The mean IBS‐SSS score for participants was 333.3 ± 89.4. Anthropometric data and clinical information were collected (Table 1).

**TABLE 1:** Demographic, clinical and lifestyle characteristics of 106 patients with IBS.

*N* = 106	Total (Mean ± SD)
	106
Age (year)	45 ± 14
BMI	26.72 ± 5.72
Duration of IBS (months)	95 ± 112
Severity of IBS (0–500)	333 ± 89
Physical Activity Level (MET‐min/wk)	3381.96 ± 4482.02
Sedentary time/day (minutes)	430.71 ± 220.59

*Note:* Scores are reported as mean ± standard deviation.

Abbreviations: BMI, body mass index; MET, metabolic equivalent of task.

### 3.2 Baseline Mental Health, Physical Activity and Quality of Life Outcomes

Fifty‐nine participants (55.7%) currently engaged in physical activity, and a total of 90 participants (84.9%) expressed willingness to engage in physical activity. Among those who were physically active 0.9% exercised once per week, 17.0% twice per week, 39.6% three times per week, and 42.5% four times or more per week. Mean sedentary time was 432.31 ± 220.15 min per day.

Participants reported significant psychological burden. Mean GAD‐7 and PHQ‐8 scores indicated mild anxiety (9.25 ± 6.38) and moderate depressive symptoms (11.11 ± 7.06). Clinically significant anxiety (GAD‐7 ≥ 10) was present in 42% percent of participants (*n* = 45), and 54% reported clinically significant depression (PHQ‐8 ≥ 10; *n* = 57).

HRQoL scores, (where higher scores denote better health status), revealed notable impairments. While Physical Functioning (60.38 ± 30.68) and Emotional Well‐being (50.57 ± 25.00) showed relatively higher mean scores, substantial deficits were evident in Energy/Fatigue (27.18 ± 22.91), Role Limitations due to Physical Health (25.95 ± 38.10), and Pain (35.09 ± 22.73). These observed HRQoL characteristics were subsequently included in correlation analyses to investigate relationships with symptom severity and other study variables, including physical activity levels, sedentary time, anxiety and depression. Comprehensive descriptive statistics for all QoL domains are provided in Table 2.

**TABLE 2:** Quality of Life (QoL) domains assessed in patients with Irritable Bowel Syndrome, showing the average scores for each domain measured using SF‐36.

*N* = 106	Total (Mean ± SD)
*QoL*	
Physical functioning (PF)	60.43 ± 30.70
Role limitation due to physical health (RL‐PH)	25.94 ± 38.10
Role limitations due to emotional health (RL‐EH)	38.99 ± 39.70
Energy/fatigue (E/F)	27.18 ± 22.91
Emotional well‐being (EWB)	50.57 ± 25.00
Social functioning (SF)	47.05 ± 27.04
Pain (P)	35.09 ± 22.73
General health (GH)	36.11 ± 18.84
Health change (HC)	44.34 ± 28.72

### 3.3 Correlations Between Study Variables

**FIGURE 2:**
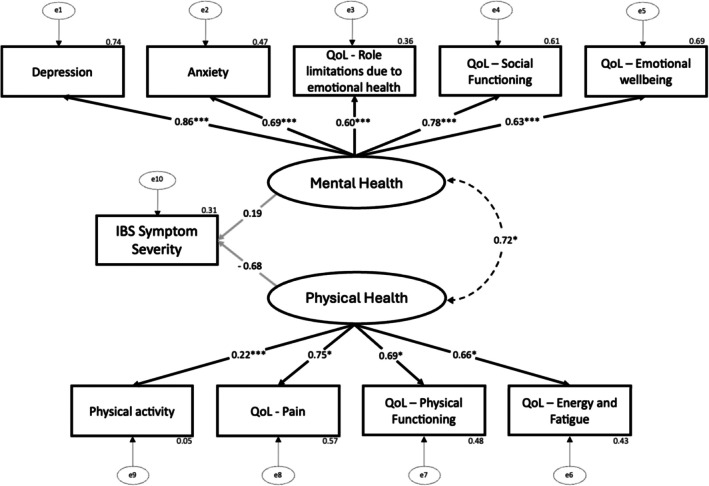
Final Path Model. Structural Equation Model (incl. all participants) showing the association between IBS symptom severity, physical health variables and mental health variables. Standardized regression coefficients (*β*) are presented along each path, representing the strength and direction of associations between variables. Black lines represent significant relationships (*p* < 0.05), gray lines represent a non‐significant relationship (*p* ≥ 0.05) and dashed lines represent correlations between parameters in the same level. Error terms (e₁–e₁₀) represent the proportion of variance in each observed variable not explained by the model. *p < 0.05; ***p < 0.001.

Correlations between IBS symptom severity, physical and mental health perceptions, QoL domains, physical activity levels, and sedentary behavior were assessed using Pearson correlation analysis. Several significant positive and negative correlations were observed between IBS symptom severity and other variables. The strongest correlation was a moderate negative association between IBS symptom severity and the QoL Pain domain (*r* = −0.454, *p* < 0.001; Table 3), indicating that greater symptom severity was associated with increased pain and poorer QoL. IBS symptom severity also showed moderate negative correlations with other HRQoL domains, including Physical Functioning (*r* = −0.341, *p* < 0.001; Table 3) and Energy/Fatigue (*r* = −0.329, *p* < 0.001; Table 3), suggesting that higher symptom burden was linked to reduced physical and emotional wellbeing. Conversely, IBS symptom severity showed positive correlations with both anxiety (*r* = 0.235, *p* < 0.05; Table 3) and depression (*r* = 0.240, p < 0.05; Table 3), reflecting the psychological impact of the condition. Physical activity levels were positively correlated with physical health domains and negatively correlated with IBS severity (Table 3). Conversely, sedentary time was negatively correlated with several QoL domains, notably Physical Functioning (*r* = −0.244, *p* < 0.05), Role Limitations due to Physical Health (*r* = −0.224, *p* < 0.05), and Pain (*r* = −0.233, *p* < 0.05). Sedentary time also positively correlated with both anxiety (*r* = 0.253, *p* < 0.01) and depression (*r* = 0.276, *p* < 0.01), indicating that increased sedentary behavior was associated with higher levels of these psychological distress measures, visually represented in Figure 2. For full correlation results see Table 3.

**TABLE 3:** Pearson Correlation Coefficients (*r*) between IBS symptoms severity, QoL domains, anxiety, depression and physical activity measures in patients with IBS.

Variable	IBS‐SSS	QoL Physical Functioning	QoL Role limitations‐ physical health	QoL Role limitations—emotional health	QoL Energy/Fatigue	QoL Emotional Wellbeing	QoL Social Functioning	QoL Pain	QoL General Health	QoL Health Change	Anxiety	Depression	GPAQ METs	Sedentary Time
IBS Symptom Severity	1													
QoL Physical Functioning	−0.341***	1												
QoL Role limitation‐physical health	−0.253***	0.351***	1											
QoL Role limitations‐ emotional health	−0.136	0.249*	0.385***	1										
QoL Energy/Fatigue	−0.329***	0.373***	0.471***	0.526***	1									
QoL Emotional Wellbeing	−0.221***	0.304**	0.182	0.485***	0.469***	1								
QoL Social Functioning	−0.266***	0.474***	0.407***	0.456***	0.456***	0.305**	1							
QoL Pain	−0.454***	0.595***	0.393***	0.242*	0.463***	0.373***	0.251*	1						
QoL General Health	−0.272***	0.457***	0.356***	0.356***	0.373***	0.251*	0.413***	0.374***	1					
QoL Health Change	−0.208**	0.278**	0.175	0.175	0.222*	0.307**	0.231*	0.175	0.175	1				
Anxiety	0.235*	−0.178	−0.178	−0.358***	−0.324***	−0.621***	−0.496***	−0.265**	−0.352***	−0.186	1			
Depression	0.240*	−0.394***	−0.412***	−0.402***	−0.300**	−0.667***	−0.399***	−0.457***	−0.457***	−0.300**	0.251*	1		
GPAQ METs	−0.068	0.308**	0.108	−0.048	0.138	0.115	0.101	0.119	0.116	−0.045	−0.095	−0.111	1	
Sedentary Time	0.153	−0.244*	−0.224*	−0.063	−0.134	−0.222*	−0.129	−0.233*	−0.250**	−0.126	0.253**	0.276**	−0.187	1

*Note:* *p < 0.05;**p < 0.01;***p < 0.001.

### 3.4 SEM Analysis

The overall model (Figure 2) explored the relationship between latent variables; mental health and physical health and observed variables. The model fit was assessed using the Tucker‐Lewis Index (TLI), Comparative Fit Index (CFI), and Root Mean Square Error of Approximation (RMSEA). When all 106 participants were included, the data demonstrated a good fit to the proposed model: *χ* ^2^ (33) = 60.513, *p* = 0.002, RMSEA = 0.089, CFI = 0.932, TLI = 0.907 [50].

### 3.5 IBS Symptom Severity

There was no significant relationship between mental health and IBS symptom severity (*β* = 0.19, 95% CI [−2.224, 8.192], *p* = 0.261). Notably, this finding does not contradict the significant bivariate correlations observed between IBS‐SSS and both anxiety and depression (Table 3), as SEM accounts for shared variance across multiple indicators and estimates relationship between latent constructs, resulting in a more conservative assessment than simple correlations. Conversely, physical health showed a large but non‐significant association with IBS symptom severity (*β* = −0.68, 95% CI [−0.126, 0.004], *p* = 0.065), suggesting that better physical health tended to be associated with lower symptom severity. Although this relationship did not reach conventional statistical significance, the magnitude and direction of the standardized coefficient indicate a potentially clinically relevant trend.

### 3.6 Physical Health

Physical health was significantly predicted by bodily pain (*β* = 0.75, 95% CI [0.001, 0.033], *p* = 0.039), physical functioning (*β* = 0.69, 95% CI [0.001, 0.041], *p* = 0.040), energy and fatigue (*β* = 0.66, 95% CI [0.001, 0.029], *p* = 0.041), and physical activity (*β* = 0.22, *p* < 0.001). These variables explained 5% of the variance in physical health (*R* ^2^ = 0.05).

### 3.7 Mental Health

Mental health was significantly predicted by anxiety (*β* = 0.69, 95% CI [0.565, 0.945], *p* < 0.001), role limitations due to emotional health (*β* = 0.60, 95% CI [2.839, 5.309], *p* < 0.001), social functioning (*β* = 0.78, 95% CI [2.870, 4.406], *p* < 0.001), emotional well‐being (*β* = 0.83, 95% CI [2.877, 4.265], *p* < 0.001), and depression (*β* = 0.86, *p* < 0.001). These variables explained a 94% proportion of the variance in mental health (*R* ^2^ = 0.94).

### 3.8 Correlation Between Mental Health and Physical Health

There was a near‐significant, strong positive correlation between mental health and physical health (*r* = 0.72, 95% CI [5.848, 8295.268], *p* = 0.050).

## 4 Discussion

The aim of this study was to identify the factors influencing IBS symptom severity in 106 clinically diagnosed IBS patients (mental health and physical health), and how these interact with each other. Based on existing literature and previously proposed connections our model hypothesized that better physical and mental health correlates with reduced IBS symptom severity.

Our SEM analysis provides valuable insights into the complex interplay of these factors. Physical health demonstrated a negative latent path coefficient to IBS symptom severity, approaching but not reaching conventional significance, while mental health did not exhibit a statistically significant direct path, suggesting its influence may be indirectly (mediated through physical health) and/or distributed across specific psychological components rather than captured as a single latent effect. Physical activity significantly predicted physical health in our study. While the path from physical health to IBS symptom severity did not reach conventional significance, the observed large association suggests that improvements in physical health could potentially influence IBS symptom severity, consistent with existing literature [51, 52]. Additionally, the significant positive correlation between latent mental and physical health constructs underscores their interrelated nature, with potential implications for clinical interventions.

### 4.1 Physical Health

The negative relationship between the latent physical health and observed IBS symptom severity suggests the clinical importance of physical functioning and activity in this population, supporting the hypothesized model (Figure 1). Although the standardized regression weight for physical health was relatively large, the association with IBS symptom severity marginally exceeded 0.05 and should therefore be interpreted cautiously. Previous research has reported that improved physical health, especially through structured exercise, correlates with lower IBS symptom severity and improved QoL [11, 12, 13, 34, 53, 54, 55], however, the present findings do not provide definitive evidence of this relationship. Clinicians may consider assessing physical health routinely in patients with moderate‐to‐severe IBS, including evaluating bodily pain, energy levels, and functional limitations as these factors can directly impact symptom burden and QoL [56]. Thus, supporting physical health and activity promotion as key adjuncts in IBS management.

Within our SEM framework, physical health was modeled as a second‐order latent construct, significantly predicted by pain, physical functioning, energy/fatigue, and physical activity. All of which are commonly disrupted in individuals with IBS, and may not only reflect the consequence of symptom burden but also act as upstream modulators of disorder experience. Assessment for overlapping DGBIs or central sensitisation conditions such as fibromyalgia may be particularly relevant in patients with high pain scores, as these comorbidities frequently co‐occur with moderate‐to‐severe IBS and contribute to worse QoL [57, 58].

Mechanistically individuals reporting lower fatigue and greater physical functioning are more likely to sustain symptom‐management behaviors such as dietary adherence, physical activity and stress regulation, factors which are documented to reduce IBS symptoms, modulate gastrointestinal symptoms and improve QoL [13, 31, 59]. Conversely, impaired physical health may restrict engagement in these self‐management behaviors and increase sedentary time, perpetuating a self‐reinforcing cycle of symptom amplification and decreased QoL (Figure 2). The interplay between these observed variables (physical activity, pain, energy/fatigue, physical functioning) may involve complex interactions between dysregulated neuroimmune pathways (altered bi‐directional communication between the brain and gastrointestinal tract), altered visceral nociception (hypersensitivity) and impaired autonomic nervous system (ANS) function contributing to altered gastrointestinal motility, all recognized contributors to IBS pathophysiology [60].

### 4.2 Mental Health

Although the direct latent path from mental health to IBS symptom severity was non‐significant in our SEM model, the strong covariance between physical and mental health latent constructs supports the well‐established link between these domains [61]. Anxiety and depression were retained as separate observed indicators within the latent mental health construct to reflect their distinct clinical presentations and measurement instruments (GAD‐7 and PHQ‐8), while allowing shared variance to be captured. As reported in the results, IBS‐SSS was positively correlated with both anxiety and depression (Table 3), consistent with prior literature [27, 29, 62]. This demonstrates that higher psychological distress is associated with greater symptom burden. The discrepancy between these bivariate correlations and the SEM path likely reflects the conservative nature of latent variable modeling, which accounts for shared variance among multiple indicators, as well as limited statistical power in our sample. Mental health was robustly predicted by anxiety, depression, emotional well‐being, social functioning, and role limitations due to emotional health, explaining 94% of variance in the latent mental health factor. While mental health did not directly predict symptom severity in this model, it likely exerts indirect influence via its interaction with physical health, through mechanisms such as fatigue, behavioral avoidance, and symptom sensitivity. Clinically, these findings underscore the importance of addressing both anxiety and depression in IBS care, not only for mental well‐being but also for its potential downstream effects on physical health and symptom severity.

These findings align with Engel's biopsychosocial model [63]; whereby psychological and behavioral factors interact to shape health outcomes [7]. For example, psychological distress may impact IBS symptom severity indirectly by influencing fatigue, behavioral avoidance, symptom sensitivity, and motivational factors. Clinically, over half the cohort reported moderate‐to‐severe depressive symptoms (54%) and clinically significant anxiety (42%), consistent with previous estimates of high psychological comorbidity in IBS populations [64, 65]. Furthermore, sedentary time correlated positively with both anxiety and depression, suggesting that inactivity may both result from and exacerbate psychological distress. These bidirectional relationships reflect complex GBA interactions where psychological comorbidity modulates symptom perception and coping behaviors [66].

### 4.3 Integration and Mechanistic Implications

The SEM results infer that mental health's influence on IBS symptoms operates predominantly through its association with physical health, suggesting that physical health acts as a proximal mediator translating psychological distress into symptomatic expression, reinforcing the need for integrated therapeutic approaches addressing both mental and physical symptomology in IBS. The observed correlations between physical and mental health (Figure 2) emphasize their mutual reinforcement and the potential for reciprocal intervention targets. Overall, these findings highlight actionable “take‐away” points for clinicians to assess both mental and physical health dimensions, identifying high‐pain or fatigued patients and considering overlapping DGBIs [57] or central sensitisation symptoms can guide integrative care strategies.

Mental health findings should also inform practice; higher anxiety and depression scores were associated with greater symptom burden, and given the bidirectional relationships between mental health, physical functioning, and activity, interventions targeting psychological distress alongside physical rehabilitation or exercise may optimize outcomes in moderate‐to‐severe IBS patients. Overall, these relationships with IBS symptom severity are consistent with previous research, supporting the validity of the observed trends despite non‐significant SEM paths.

### 4.4 Strengths and Limitations

This study's strengths include the application of SEM to model latent constructs, allowing for a more complex understanding of how multiple interrelated domains contribute to multifactorial conditions like IBS. The use of a clinically diagnosed cohort conforming to Rome IV criteria and the consistent application of validated questionnaires for all measured constructs enhances the internal validity of our findings by reducing diagnostic misclassification which can occur with self‐reported IBS in the general population. However, this recruitment approach also limited access to individuals with milder symptom profiles, resulting in an overrepresentation of moderate‐to‐severe IBS and potentially limiting generalisability to milder IBS cases.

However, several limitations should be acknowledged. The cross‐sectional design limits the ability to determine causality; therefore, longitudinal research is needed to establish directionality. Additionally, the reliance on self‐reported measures, particularly for physical activity and sedentary behavior, may introduce measurement bias, including inaccuracies related to recall and the influence of social desirability. Specifically, while physical activity (METS) predicted physical health in our model, the study was not designed or powered to identify specific MET thresholds associated with higher IBS symptom severity; thus, future studies with larger samples are needed to explore the dose–response relationships between physical activity and IBS‐SSS, incorporating objective assessment methods, such as accelerometry to enhance data validity.

Although the sample size (*N* = 106) supported adequate model fit, certain paths such as physical health to IBS‐SSS, approached but did not reach statistical significance, indicating that larger samples may yield greater power. Additionally, IBS is a highly heterogenous disorder and while SEM provides a robust framework for modeling complex interrelationships, it may be limited in its ability to capture subtype‐specific or individual‐level pathways in smaller samples. Larger cohorts may permit stratified analyses by IBS subtype (e.g., IBS‐D, IBS‐C, IBS‐M), allowing exploration of potential subtype‐specific pathways. Accordingly, the findings should be interpreted as exploratory and hypothesis‐generating rather than confirmatory. Additionally, health‐related QoL measures such as the SF‐36 may be influenced by comorbid conditions common in chronic conditions and IBS populations, which could confound interpretations of physical health perception and may factors beyond the primary diagnosis rather than IBS‐specific impairment. Finally, the single‐center recruitment and the predominantly female cohort limit the generalisability of these findings to broader and more diverse IBS populations, including males and those in different healthcare settings.

### 4.5 Clinical Implications

Despite these limitations, our findings offer important clinical implications. The association between physical health and IBS symptom severity, coupled with the link between mental and physical health, strongly supports advocating for multi‐modal and comprehensive IBS management. For example, physical activity may be considered not just as general health advice, but as a potential therapeutic adjunct for IBS. Given the high prevalence of psychological distress and its association with sedentary behavior and various QoL domains, routine screening for anxiety and depression, along with integrated psychological support, remains paramount in IBS care. A rounded, patient‐centered approach that actively addresses and integrates both physical activity and mental well‐being is crucial for optimizing overall QoL and potentially alleviating symptom burden in IBS.

### 4.6 Future Research Directions

Future research should build upon these findings by employing longitudinal designs to definitively establish the directionality and causality of the observed relationships. Intervention studies specifically designed to increase physical activity and reduce sedentary time in IBS patients are crucial to evaluate their direct impact on IBS symptom severity, mental health, and QoL. Such studies could also explore the specific types, intensities, and durations of physical activity most beneficial for this population. Further investigation into potential mediating variables that link psychological factors and physical health to IBS outcomes could provide a deeper understanding of the complex underlying mechanisms, including specific GBA pathways. Finally, replicating these findings in larger, more diverse cohorts would strengthen the generalisability of the results and illuminate any subgroup‐specific considerations.

## 5 Conclusions

Using Structural Equation Modeling, this study suggests that better physical health is associated with lower IBS symptom severity. Physical health, characterized by pain levels, physical functioning, energy/fatigue, and physical activity, serves as a critical mediator in symptom expression. In contrast, mental health, though strongly correlated with physical health, did not directly predict IBS symptom severity in this model. Instead, mental health may influence symptoms indirectly via its impact on physical health behaviors, such as activity engagement, fatigue perception, and motivation for symptom management. This suggests that mental health impacts IBS indirectly through behavioral and physiological pathways, thus underscoring the need for integrated care that addresses both physical and mental health concurrently in IBS, alongside conventional pharmacological interventions. This multifaceted approach is crucial to improve symptom control, improve QoL, and break the cyclical interaction between mental health and physical health and symptom exacerbation in IBS.

Future longitudinal studies and targeted interventions are essential to establish causal pathways, determine optimal physical activity prescriptions, and develop tailored therapies that consider sex differences and IBS subtypes to comprehensively address the complex biopsychosocial mechanisms underlying IBS.

## Author Contributions

H.B.L. coordinated the study, recruited participants, and wrote the manuscript. A.M.D. and M.C. made substantial contributions to participant recruitment. A.M. was responsible for data entry into the NHS database. G.E.W. contributed to the study design. N.C.W., D.M., and M.C. provided critical revision of the manuscript. All authors reviewed and approved the final manuscript.

## Funding

The authors have nothing to report.

## Conflicts of Interest

The authors declare no conflicts of interest.

## Data Availability

The data that support the findings of this study are available from the corresponding author upon reasonable request.
